# Supplementary material for: RNAPII-dependent ATM signaling at collisions with replication forks
Source: Nat Commun. 2023 Aug 24;14:5147. doi: 10.1038/s41467-023-40924-4 (PMC10449895; doi:10.1038/s41467-023-40924-4)
Supplement: Supplementary file 1 — Supplementary Information [file 41467_2023_40924_MOESM1_ESM.pdf]

Supplementary material
RNAPII-dependent ATM signaling at collisions with replication forks

Elias Einig[1], Chao Jin[1], Valentina Andrioletti[1,2], Boris Macek[3], Nikita Popov[1*]

1 Department of Medical Oncology and Pulmonology, University Hospital Tübingen, Otfried-Mueller-Str 14, 72076 Tübingen, Germany
2 Present address: enGenome S.R.L., Via Fratelli Cuzio 42, 27100 Pavia, Italy
3 Interfaculty Institute of Cell Biology, Eberhard Karls University of Tübingen, Auf d. Morgenstelle 15, 72076 Tübingen, Germany

* Correspondence: nikita.popov@med.uni-tuebingen.de

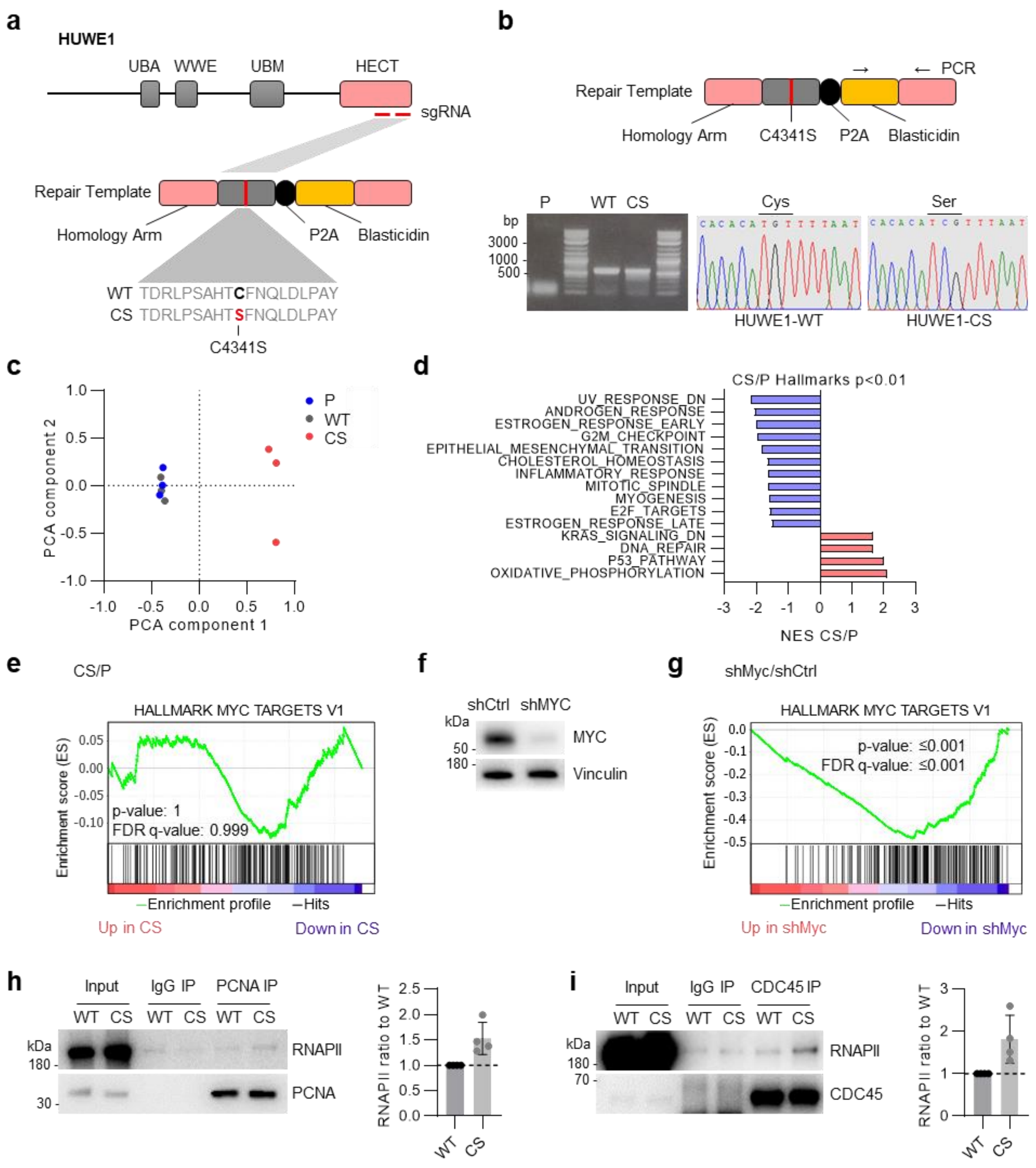

Supplementary Figure 1: Mutation of HUWE1 induces transcription-replication conflicts

a) Gene replacement strategy to exchange the catalytic cysteine of endogenous HUWE1 with either a serine (CS) or cysteine (WT) in HCT116 cells (P). Specific sgRNAs targeting the HUWE1 HECT domain were used to cleave genomic DNA and a repair template encoding the flanking sequences, the P2A sequence and a blasticidin resistance gene was provided. **b)** Validation of successful gene replacement by PCR and Sanger sequencing of the respective gene locus (n=2). **c)** Principal component analysis of RNAseq experiments (n=3). **d)** Enriched hallmark gene sets resulting from GSEA[1] analysis of CS/P cells. Normalized enrichment scores (NES) and nominal p-values were derived from two-tailed t-test and adjusted for multiple testing against all hallmark gene sets. **e)** Gene set enrichment analysis (GSEA) for hallmark MYC targets in CS cells compared with P cells. Nominal p value is derived from two-tailed t-test without adjustment since only one gene set was tested. **f)** Immunoblots of HCT116 cells expressing shMYC or shCtrl (n=2). **g)** GSEA for hallmark MYC targets in HCT116 cells expressing shMYC or shCtrl. Nominal p value is derived from two-tailed t-test without adjustment since only one gene set was tested. **h,i)** Immunoprecipitation (IP) of PCNA (h) or CDC45 (i) in formaldehyde-

crosslinked and sonicated HUWE1-WT and HUWE1-CS cells. A representative image (left) and quantification of the CS/WT ratio (right) are shown (n=4, mean±SD).
Source data are provided as a Source Data file.

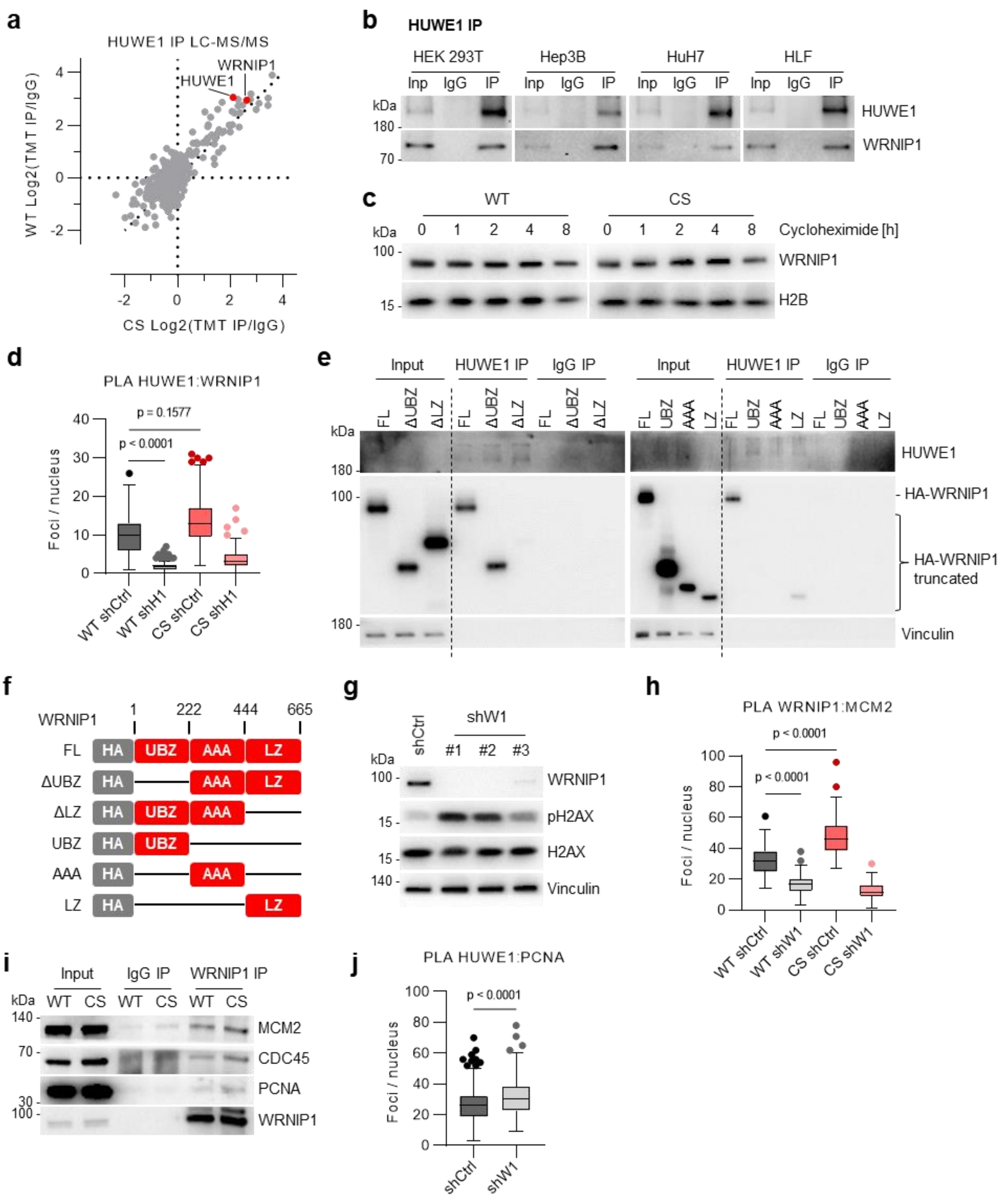

Supplementary Figure 2: WRNIP1 binds HUWE1 via its leucine zipper domain to suppress TRCs. **a)** Immunoprecipitation of HUWE1 followed by tandem mass tag (TMT) labeling and LC-MS/MS analysis in HUWE1-WT and HUWE1-CS cells. **b)** Immunoprecipitation (IP) of HUWE1 followed by immunoblotting with HUWE1 and WRNIP1 antibodies (n=2). **c)** Cycloheximide assay in HUWE1-WT and HUWE1-CS cells (n=2). **d)** PLA assay with HUWE1 and WRNIP1 antibodies in WT and CS cells expressing a control (shCtrl) or HUWE1-targeting (shH1) shRNAs (n=76,111,85,122 cells). **e)** Immunoprecipitation of HUWE1 from cells expressing HA-tagged WRNIP1 truncation variants (n=3). **f)** Schematic of WRNIP1 truncation variants. FL: Full length; UBZ: Ubiquitin binding zinc finger; LZ: Leucine zipper; AAA: AAA+ ATPase domain. **g)** Expression of three different shRNAs against WRNIP1 (shW1) followed by immunoblot analysis. **h)** PLA with antibodies to WRNIP1 and MCM2 in HUWE1-WT and HUWE1-CS cells, expressing shCtrl or shWRNIP1 (n=128,85,82,106 cells). **i)** Immunoprecipitation of WRNIP1 in lysates of HUWE-WT and HUWE1-CS cells followed by immunoblotting. **j)** PLA with antibodies to HUWE1 and PCNA in shWRNIP1 and shCtrl cells (n=403,322 cells). P-values were

determined using the non-parametric, two-tailed Mann-Whitney test. **d,h)** Boxplots show median±quartiles with whiskers ranging up to 1.5-fold of the inter-quartile range. P-values were determined using Kruskal-Wallis test followed by Dunn‘s multiple comparison.
Source data are provided as a Source Data file.

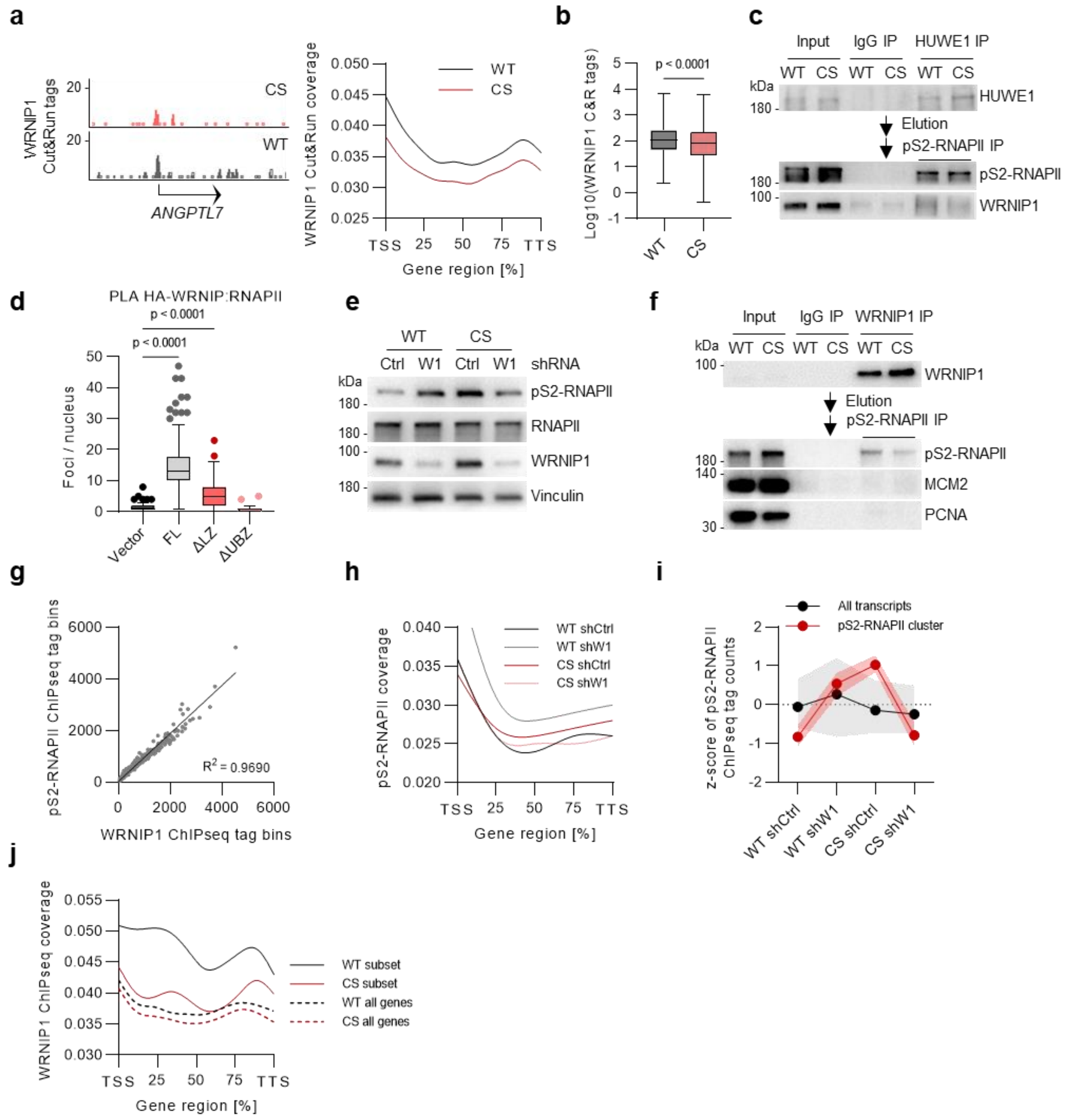

Supplementary Figure 3: HUWE1 controls association of WRNIP1 with elongating RNAPII

a) Example track of Cut&Run analysis using WRNIP1 antibodies in HUWE1-WT and HUWE1-CS cells (left). WRNIP1 Cut&Run tags were quantified at genic regions of all genes and displayed as scaled metagenes (right). **b)** WRNIP1 C&R tag bins for all genes in HUWE1-WT and HUWE1-CS cells. n=4646 bins of 10 gene regions each. Significance was determined using a paired t-test. Boxplots represent median±quartiles with whiskers ranging from minimum to maximum values. **c)** Sequential immunoprecipitation (IP) of HUWE1 followed by elution and subsequent immunoprecipitation with pS2-RNAPII antibodies in formaldehyde-crosslinked and sonicated HUWE1-WT and HUWE1-CS cells (n=2). **d)** PLA with antibodies against RNAPII and HA-tag in cells expressing the indicated HA-tagged WRNIP1 truncation variants. Boxplots show median±quartiles with whiskers ranging up to 1.5-fold of the inter-quartile range (n=143,236,142,133 cells). P-values were determined using Kruskal-Wallis test followed by Dunn‘s multiple comparison. FL: Full length; UBZ: Ubiquitin binding zinc finger; LZ: Leucine zipper. **e)** Immunoblots of HUWE1-WT and HUWE1-CS cells expressing shCtrl or shWRNIP1 (n=3). **f)** Sequential immunoprecipitation of WRNIP1 followed by elution and subsequent immunoprecipitation with pS2-RNAPII antibodies in formaldehyde-crosslinked and sonicated HUWE1-WT and HUWE1-CS cells. l. exp.: long exposure (n=2). **g)** Correlation of WRNIP1 and pS2-RNAPII or RNAPII ChIPseq tags. Each dot represents an averaged bin of 10 values sorted by WRNIP1 tag counts. **h)** Metagene analysis

of pS2-RNAPII ChIPseq data at gene bodies of all genes. **i)** Z-score based clustering of pS2-RNAPII ChIPseq tags for gene areas with the most pronounced HUWE1- and WRNIP1-dependent pS2-RNAPII accumulation, which was reverted in the double mutant. Dots represent median values with error bands of the 25th and 75th percentile. **j)** ChIPseq analysis of WRNIP1 at gene regions for a subset of 4643 genes with the most pronounced HUWE1- and WRNIP1-dependent pS2-RNAPII accumulation shown in i).
Source data are provided as a Source Data file.

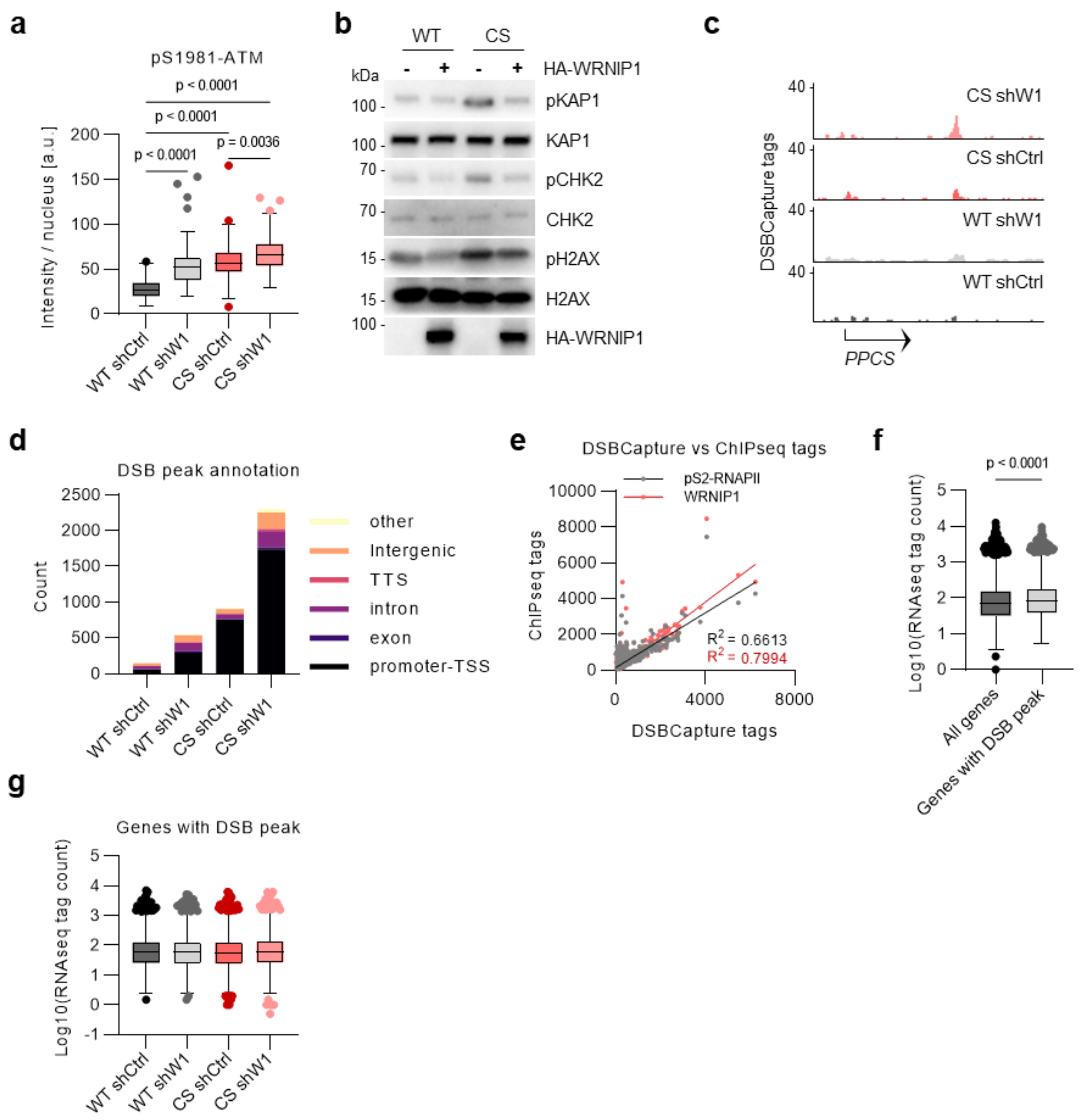

Supplementary Figure 4: HUWE1 and WRNIP1 suppress ATM signaling.
a) Immunofluorescence intensity of pS1981-ATM in HUWE-WT or HUWE1-CS cells expressing shWRNIP1 or shCtrl. From left, n=165,105,162,144 cells. Boxplots show median±quartiles with whiskers ranging up to 1.5 fold of the inter-quartile range. P-values were determined using Kruskal-Wallis test followed by Dunn‘s multiple comparison. a.u.: arbitrary units. **b)** HA-WRNIP1 overexpression in HUWE1-WT or HUWE1-CS cells followed by western blot analysis (n=2). **c)** Representative genome browser tracks of DSBCapture tags in HUWE1-WT and HUWE1-CS cells expressing shWRNIP1 or shCtrl. See also Figure 4C. **d)** Annotation of DSBCapture peaks. **e)** Correlation of WRNIP1 and pS2-RNAPII ChIPseq tags with DSBCapture signal for gene areas of genes with a DSB peak. **f)** RNAseq tag counts for all genes and genes with a DSB peak. Significance was determined using an unpaired, two-tailed t-test. From left, n=11350,2902 gene regions. **g)** RNAseq tag counts for genes with a DSB peak in the indicated cell lines. n=2855 genes per group in two biological replicates. **a,f,g)** Boxplots show median±quartiles with whiskers ranging up to 1.5 fold of the inter-quartile range.
Source data are provided as a Source Data file.

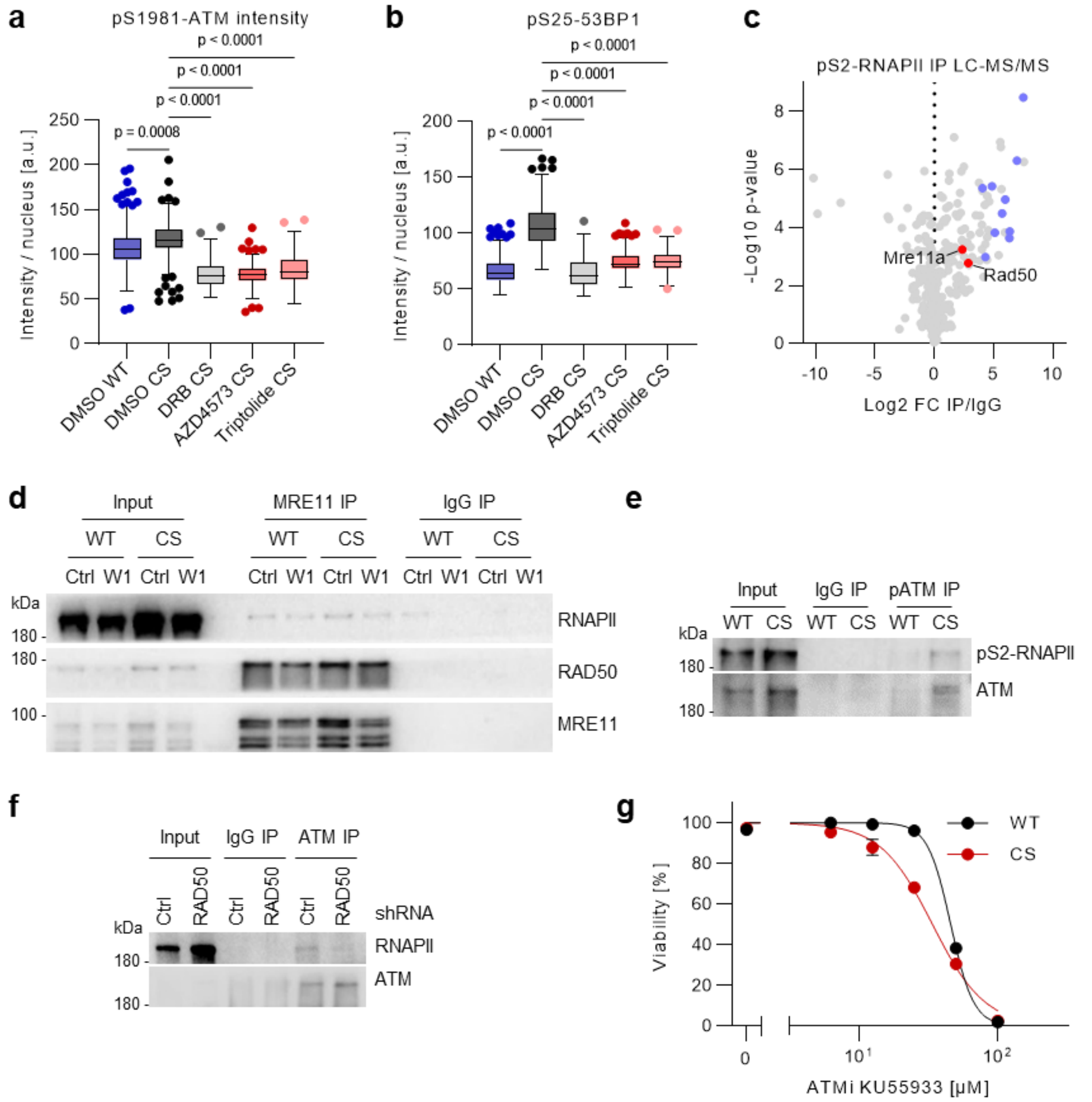

Supplementary Figure 5: HUWE1 and WRNIP1 limit ATM activation at RNAPII.

a) Immunofluorescence staining of pS1981-ATM in HUWE1-WT cells or HUWE1-CS cells treated with 33 nM AZD4573, 33 nM triptolide or 50 µM DRB for 5 h. From left, n=256,250,228,188,211 cells. Boxplots show median±quartiles with whiskers ranging up to 1.5 fold of the inter-quartile range. P-values were determined using Kruskal-Wallis test followed by Dunn's multiple comparison. **b)** Immunofluorescence staining of pS25-53BP1 in HUWE1-WT cells or HUWE1-CS cells treated and analyzed as in a). From left, n=256,250,228,188,211 cells **c)** Immunoprecipitation of pS2-RNAPII in benzonase-treated lysates of mouse embryonic fibroblasts (MEFs) followed by LC-MS/MS analysis. Subunits of the RNAPII complex are highlighted in blue. **d)** Immunoprecipitation (IP) of MRE11 in HUWE1-WT and HUWE1-CS cells, expressing shCtrl or shWRNIP1. **e)** Immunoprecipitation of pS1981-ATM in HUWE1-WT and HUWE1-CS cells. **f)** Immunoprecipitation of ATM in benzonase-treated lysates of HUWE1-CS cells expressing shRAD50 or shCtrl. **g)** Dose-response curve of HUWE1-WT and HUWE1-CS cells treated with the ATM inhibitor KU55933 for 4 days. Cell viability was determined by WST-8 assay (n=3).

Source data are provided as a Source Data file.

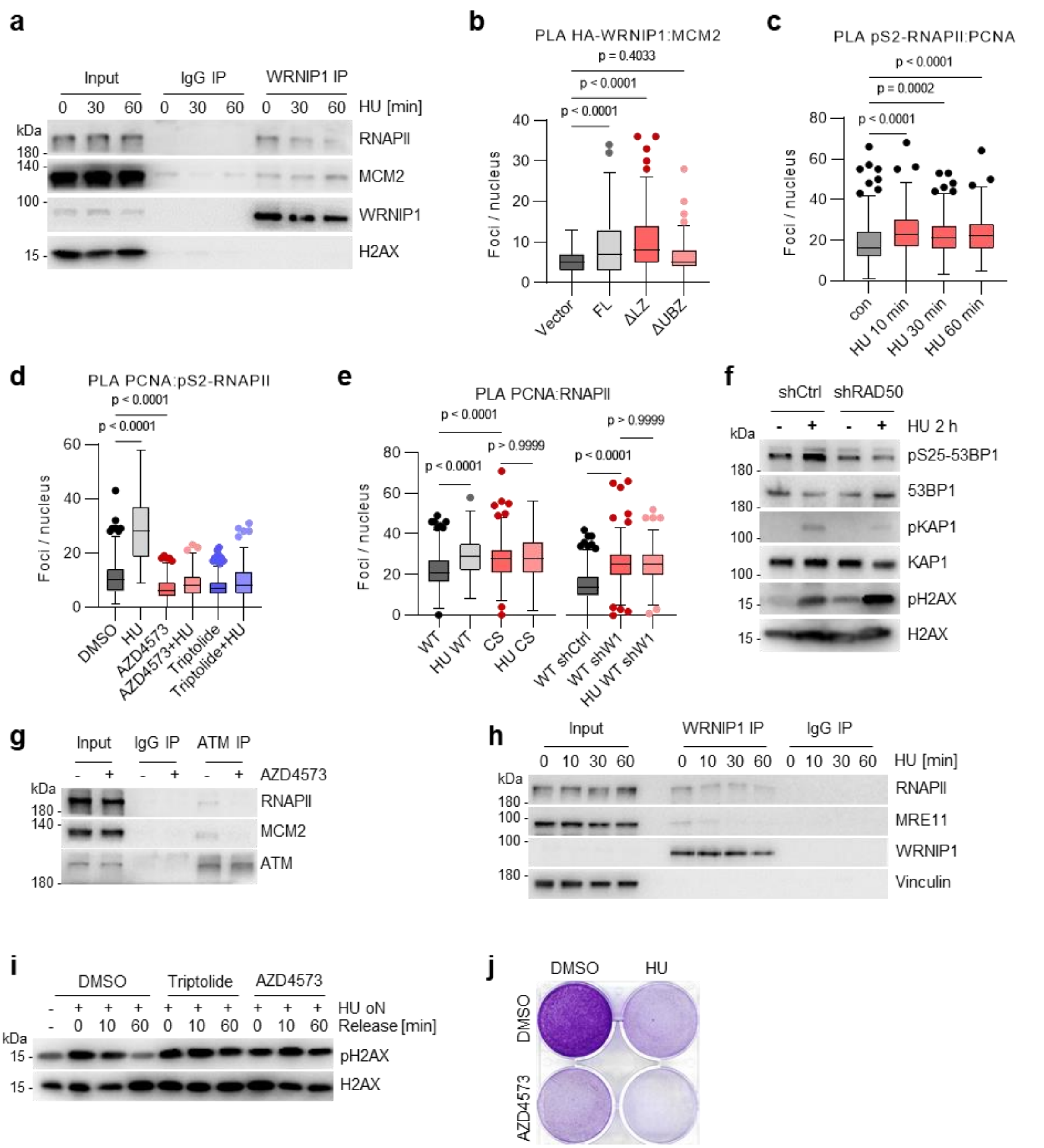

Supplementary Figure 6: HUWE1 and WRNIP1 control RNAPII-dependent ATM activation upon replicative stress.

a) Immunoprecipitation (IP) of WRNIP1 in cells treated with 1 mM hydroxyurea (HU) for the indicated time. **b)** PLA with antibodies to HA-tag and MCM2 in HCT116 cells, expressing HA-tagged WRNIP1 variants (n=117,162,181,149 cells). **c)** PLA with antibodies to PCNA and pS2-RNAPII in cells treated with 1 mM hydroxyurea (HU) for the indicated time (n=224,217,215,238,241,193 cells). **d)** PLA with antibodies to PCNA and pS2-RNAPII in cells treated with 20 nM AZD4573 or 100 nM triptolide for 2 h followed by addition of 1 mM HU for 4 h (n=199,149,177,232,203,213 cells). **e)** PLA with antibodies to PCNA and RNAPII in HUWE1-WT, HUWE1-CS and shWRNIP1 (shW1) cells, treated with DMSO or with 1 mM HU for 2 h (n=181,183,173,130;349,206,111 cells). **f)** Immunoblot of HCT116 cells expressing shCtrl or shRAD50 treated with 1 mM HU for 2 h. **g)** Immunoprecipitation of ATM from benzonase-treated lysates of cells treated with 1 mM HU with or without 20 nM AZD4573 for 4 h. **h)** Immunoprecipitation of WRNIP1 from HCT116 cells, treated with 1 mM hydroxyurea (HU) for the indicated time. **i)** Immunoblots of HCT116 cells, treated with 1 mM HU alone or in combination with 100 nM triptolide or with 20 nM AZD4573 overnight, followed by release for the indicated time. **j)** Crystal violet staining of HeLa cells, treated with 1 nM AZD4573 or 2.5 nM triptolide for 3 h, followed by addition

of 250 µM HU for 4 days. **b,c,d,e)** Boxplots show median±quartiles with whiskers ranging up to 1.5-fold of the inter-quartile range. P-values were determined using Kruskal-Wallis test followed by Dunn's multiple comparison.
Source data are provided as a Source Data file.

Supplementary References

1. Subramanian A*, et al.* Gene set enrichment analysis: a knowledge-based approach for interpreting genome-wide expression profiles. *Proc Natl Acad Sci U S A* **102**, 15545-15550 (2005).
