# Supplementary material for: RNAPII-dependent ATM signaling at collisions with replication forks
Source: Nat Commun. 2023 Aug 24;14:5147. doi: 10.1038/s41467-023-40924-4 (PMC10449895; doi:10.1038/s41467-023-40924-4)
Supplement: Supplementary file 3 — Description of Additional Supplementary Files [file 41467_2023_40924_MOESM3_ESM.pdf]

Description of Additional Supplementary Files Document

File Name: Supplementary Data 1
Description: List of commercial reagents. This file contains a list of catalog numbers and company names of commercial reagents used throughout this study
