# Supplementary material for: RNAPII-dependent ATM signaling at collisions with replication forks
Source: Nat Commun. 2023 Aug 24;14:5147. doi: 10.1038/s41467-023-40924-4 (PMC10449895; doi:10.1038/s41467-023-40924-4)
Supplement: Supplementary file 5 — Reporting Summary [file 41467_2023_40924_MOESM5_ESM.pdf]

nature portfolio

Corresponding author(s): Nikita Popov, nikita.popov@med.uni-tuebingen.de

Last updated by author(s): Jan 8, 2023

Reporting Summary

Nature Portfolio wishes to improve the reproducibility of the work that we publish. This form provides structure for consistency and transparency in reporting. For further information on Nature Portfolio policies, see our Editorial Policies and the Editorial Policy Checklist.

Statistics

For all statistical analyses, confirm that the following items are present in the figure legend, table legend, main text, or Methods section.

n/a	Confirmed	
☐	☒	The exact sample size (*n*) for each experimental group/condition, given as a discrete number and unit of measurement
☐	☒	A statement on whether measurements were taken from distinct samples or whether the same sample was measured repeatedly
☐	☒	The statistical test(s) used AND whether they are one- or two-sided<br>*Only common tests should be described solely by name; describe more complex techniques in the Methods section.*
☒	☐	A description of all covariates tested
☐	☒	A description of any assumptions or corrections, such as tests of normality and adjustment for multiple comparisons
☐	☒	A full description of the statistical parameters including central tendency (e.g. means) or other basic estimates (e.g. regression coefficient) AND variation (e.g. standard deviation) or associated estimates of uncertainty (e.g. confidence intervals)
☐	☒	For null hypothesis testing, the test statistic (e.g. *F*, *t*, *r*) with confidence intervals, effect sizes, degrees of freedom and *P* value noted<br>*Give P values as exact values whenever suitable.*
☒	☐	For Bayesian analysis, information on the choice of priors and Markov chain Monte Carlo settings
☒	☐	For hierarchical and complex designs, identification of the appropriate level for tests and full reporting of outcomes
☒	☐	Estimates of effect sizes (e.g. Cohen's *d*, Pearson's *r*), indicating how they were calculated

Our web collection on statistics for biologists contains articles on many of the points above.

Software and code

Policy information about availability of computer code

Data collection	Q Exactive™ HFX Hybrid Quadrupole-Orbitrap™ mass spectrometer (Thermo Xcalibur™ version 4.3), Illumina NovaSeq 6000 (NovaSeq control software v1.7 and v1.8), Olympus BX63 microscope (cellSens Dimension version 1.17), Biorad ChemiDoc MP imaging system (ImageLab version 5.2.1)
Data analysis	NGS data: Illumina bcl2fastq (version 2.20), Skewer (version 0.2.2), STAR (version 2.5.4), EdgeR (version 3.26.8), Homer (version 4.10.3), MAGECK (0.5.9).<br>Mass spectrometry data: MaxQuant (version 1.6.17.0 or version 2.0.3.0), Perseus (version 1.6.14.0)<br>Immunofluorescence data: FIJI/ImageJ (version 1.53f)

For manuscripts utilizing custom algorithms or software that are central to the research but not yet described in published literature, software must be made available to editors and reviewers. We strongly encourage code deposition in a community repository (e.g. GitHub). See the Nature Portfolio guidelines for submitting code & software for further information.

Data

Policy information about availability of data

All manuscripts must include a data availability statement. This statement should provide the following information, where applicable:

- Accession codes, unique identifiers, or web links for publicly available datasets
- A description of any restrictions on data availability
- For clinical datasets or third party data, please ensure that the statement adheres to our policy

All data and reagents are available from the corresponding author upon request. The source data generated in this study are provided in the Supplementary Information and the Source Data file.
The NGS data generated in this study have been deposited in the GEO database under GEO series accession number GSE218240 [https://www.ncbi.nlm.nih.gov/geo/query/acc.cgi?acc=GSE218240].
The mass spectrometry data generated in this study have been deposited in the PRIDE database with the dataset identifier PXD037677 [https://www.ebi.ac.uk/pride/archive/projects/PXD037677], PXD037680 [https://www.ebi.ac.uk/pride/archive/projects/PXD037680] and PXD037812 [https://www.ebi.ac.uk/pride/archive/projects/PXD037812].
Mass spectra were searched against UniProt reference proteome databases for Mus musculus (release_2020-10-07) or Homo sapiens (release_2019-12-11) [https://www.uniprot.org/] with the Andromeda search engine.

Human research participants

Policy information about studies involving human research participants and Sex and Gender in Research.

Reporting on sex and gender	N/A
Population characteristics	N/A
Recruitment	N/A
Ethics oversight	N/A

Note that full information on the approval of the study protocol must also be provided in the manuscript.

Field-specific reporting

Please select the one below that is the best fit for your research. If you are not sure, read the appropriate sections before making your selection.

☒ Life sciences ☐ Behavioural & social sciences ☐ Ecological, evolutionary & environmental sciences

For a reference copy of the document with all sections, see nature.com/documents/nr-reporting-summary-flat.pdf

Life sciences study design

All studies must disclose on these points even when the disclosure is negative.

Sample size	Cell culture based experiments had variable sample sizes, ranging from 50 to 400 cells or DNA fibers analyzed per sample, as shown in the figure legends. In general, key experiments were replicated more than three times and sample sizes were chosen similar to previous publications: Porebski, iScience (https://doi.org/10.1016/j.isci.2019.10.010); Lemaçon, Nat. Comm. (https://doi.org/10.1038/s41467-017-01180-5); Mijic, Nat Comm. (https://doi.org/10.1038/s41467-017-01164-5).
Data exclusions	No data was excluded from analysis, except for the analysis of immunofluorescence images of proximity ligation assays. Here, overlapping nuclei and nuclei at the image borders were excluded from the analysis since these events do not represent an entire single cell.
Replication	Key experiments were replicated at least three times
Randomization	Sample allocation was not randomized since in most experiments different genotypes of cell culture samples were compared with each other. Cells were seeded prior to any treatment or molecular assay. This selection of a random subpopulation derived from an asynchronous bulk population may be regarded as an intrinsic source of randomization that is inherent to any cell culture-based experiment. During analysis, individual cells, fibers or lysates of thousands of cells were analyzed. Therefore, management of covariates was not necessary for these analyses. Immunofluorescence images were acquired at random positions of the respective microscopy slide.
Blinding	Researchers were not blinded during group allocation, data collection and analysis due to the unbiased nature of molecular assays and respective machine-derived measurements. Analysis of Image data and sequencing data was software-based.

Reporting for specific materials, systems and methods

We require information from authors about some types of materials, experimental systems and methods used in many studies. Here, indicate whether each material, system or method listed is relevant to your study. If you are not sure if a list item applies to your research, read the appropriate section before selecting a response.

Materials & experimental systems

n/a	Involved in the study
☐	☒ Antibodies
☐	☒ Eukaryotic cell lines
☒	☐ Palaeontology and archaeology
☒	☐ Animals and other organisms
☒	☐ Clinical data
☒	☐ Dual use research of concern

Methods

n/a	Involved in the study
☐	☒ ChIP-seq
☒	☐ Flow cytometry
☒	☐ MRI-based neuroimaging

Antibodies

Antibodies used

Primary antibodies (dilution for western blot 1:1000; for PLA and immunofluorescence imaging 1:200): 53BP1 4937 Cell Signaling; ATM sc-377293 Santa Cruz; ATR 2790 Cell Signaling; BrdU BD347580 BD; BrdU ab6326 Abcam; CDC45 11881S Cell Signaling; CHK2 2662T Cell Signaling; H2AX sc-517336 Santa Cruz; HA-tag 2367 Cell Signaling; HA-tag 3724 Cell Signaling; Histone H2B 2934 Cell Signaling; Histone H3 96C10S Cell Signaling; HUWE1 A300-486A-A Bethyl; KAP1 15202-1-AP Proteintech; MCM2 3619S Cell Signaling; MCM5 ab17967 Abcam; MRE11 NB100-142 Novus Biologicals; MRE11 sc-135992 Santa Cruz; MYC 13987 Cell Signaling; PCNA sc-56 Santa Cruz; pS139-H2AX 9718 Cell Signaling; pS139-H2AX 517348 Santa Cruz; pS1981-ATM 47739 Santa Cruz; pS1981 ATM ab81292 Abcam; pS2-RNAPII 61083 Active Motif; pS2-RNAPII A300-654A-M Bethyl; pS25-53BP1 sc-135748 Santa Cruz; pS4/8-RPA2 54762S Cell Signaling; pS428-ATR 2853T Cell Signaling; pS5-RNAPII 39749 Active Motif; pS824-KAP1 ab243870 Abcam; pT68-CHK2 ab3501 Abcam; RAD50 sc-74460 Santa Cruz; RAD50 NB100-154 Novus Biologicals; RBP1 (RNAPII) 14958 Cell Signaling; RNAPII 101307 Active Motif; RPA2 sc-56770 Santa Cruz; Vinculin V9131 Sigma; WRNIP1 377402 Santa Cruz; WRNIP1 A301-389A-T Bethyl;

Secondary antibodies (dilution for western blot 1:7500; for PLA and immunofluorescence imaging 1:200): Anti-mouse IgG (Alexa Fluor® 488 Conjugate) 4408 Cell Signaling; Anti-mouse IgG (Alexa Fluor® 555 Conjugate) 4409 Cell Signaling; Anti-rabbit IgG (Alexa Fluor® 488 Conjugate) 4412 Cell Signaling; Anti-rabbit IgG (Alexa Fluor® 555 Conjugate) 4413 Cell Signaling; Anti-rat IgG (Alexa Fluor® 488 Conjugate) 4416 Cell Signaling; Anti-rabbit IgG, HRP-linked Antibody 7074 Cell Signaling; Anti-mouse IgG, HRP-linked Antibody 7076 Cell Signaling; Anti-rat IgG, HRP-linked Antibody 7077 Cell Signaling;

For ChIPseq experiments 1 - 2 µg of the following antibodies were used: WRNIP1 (G-2) Santa Cruz Cat# 377402 Lot# l1516; pS2-RNAPII Active Motif Cat# 61083 Lot# 32820004

Validation

Antibodies were validated by the manufacturer (see below) or using shRNA mediated knockdown of target proteins.
53BP1 4937 Cell Signaling Supplier validation: Reactivity - H Mk Application - WB, IHC, IF ;
ATM (G-12) sc-377293 Santa Cruz Supplier validation: Reactivity - H Application - WB, IP, IF, IHC(P) and ELISA ;
ATR 2790 Cell Signaling Supplier validation: Reactivity - H Mk Application - WB ;
BrdU (B44) BD347580 BD Supplier validation: Reactivity - Species independent Application - Flow cytometry, Intracellular staining (flow cytometry) ;
BrdU [BU1/75 (ICR1)] ab6326 Abcam Supplier validation: Reactivity - Species independent Application - ICC/IF, Flow Cyt (Intra), IHC-P ;
CDC45 11881S Cell Signaling Supplier validation: Reactivity - H M R Mk Application - WB, IP, IF ;
CHK2 2662T Cell Signaling Supplier validation: Reactivity - H M R Mk Application - WB, IP ;
H2AX (938CT5.1.1) sc-517336 Santa Cruz Supplier validation: Reactivity - H Application - WB, IP, IF and IHC(P) ;
HA-tag (6E2) 2367 Cell Signaling Supplier validation: Reactivity - Species independent Application - WB, IHC, IF, F ;
HA-tag (C29F4) 3724 Cell Signaling Supplier validation: Reactivity - Species independent Application - WB, IP, IHC, IF, F, ChIP ;
Histone H2B (53H3) 2934 Cell Signaling Supplier validation: Reactivity - H M R Mk Z Application - WB ;
Histone H3 (96C10) 3638 Cell Signaling Supplier validation: Reactivity - H M R Mk Application - WB ;
HUWE1 A300-486A-A Bethyl Supplier validation: Reactivity - H Application - WB, IP, IHC ;
KAP1 15202-1-AP Proteintech Supplier validation: Reactivity - H M R Application - WB, IP, IHC, IF, FC, ChIP, ELISA ;
MCM2 (D7G11) 3619S Cell Signaling Supplier validation: Reactivity - H M R Mk Application - WB, IP, IHC, IF, ChIP ;
MCM5 ab17967 Abcam Supplier validation: Reactivity - H Application - WB, IHC-P ;
MRE11 NB100-142 Novus Biologicals Supplier validation: Reactivity - Hu, Mu, Rt, Ch, Ha, Bt, Bv, Ca, Pm, Eq, Fe, Pm Application - WB, Simple Western, ChIP, ELISA, Flow, IB, ICC/IF, IHC, IHC-Fr, IHC-P, IP, PLA, KD, KO ;
MRE11 (18) sc-135992 Santa Cruz Supplier validation: Reactivity - H M R Application - WB, IP, IF ;
MYC (D3N8F) 13987 Cell Signaling Supplier validation: Reactivity - H M R Mk Application - WB, IF, F, ChIP, C&R ;
PCNA (PC10) sc-56 Santa Cruz Supplier validation: Reactivity - H M R Y Application - WB, IP, IF, IHC(P) and FCM ;
pS139-H2AX (20E3) 9718 Cell Signaling Supplier validation: Reactivity - H M R Mk Application - WB, IHC, IF, F ;
pS139-H2AX 517348 Santa Cruz Supplier validation: Reactivity - H M R Application - WB, IP and IF ;
pS1981-ATM (10H11.E12) 47739 Santa Cruz Supplier validation: Reactivity - H M R Application - WB, IP, IF and IHC(P) ;
pS1981 ATM (EP1890Y) ab81292 Abcam Supplier validation: Reactivity - H Application - Dot blot, Flow Cyt (Intra), WB, IHC-P, IP ;
pS2-RNAPII 61083 Active Motif Supplier validation: Reactivity - H M Application - WB, IP, IF, ChIP, ICC ;
pS2-RNAPII A300-654A-M Bethyl Supplier validation: Reactivity - H M Application - WB, IP, IHC ;
pS25-53BP1 (38.Ser 25) sc-135748 Santa Cruz Supplier validation: Reactivity - H M Application - WB, IP and ELISA ;

March 2021

pS4/8-RPA2 (E5A2F) 54762S Cell Signaling Supplier validation: Reactivity - H Application - WB, IF, F ;
pS428-ATR 2853T Cell Signaling Supplier validation: Reactivity - H M R Mk Application - WB ;
pS5-RNAPII 39749 Active Motif Supplier validation: Reactivity - H M Application - WB, ChIP ;
pS824-KAP1 (BL-246-7B5) ab243870 Abcam Supplier validation: Reactivity - H M Application - IP, IHC-P, WB, ICC/IF ;
pT68-CHK2 ab3501 Abcam Supplier validation: Reactivity - H Application - WB ;
RAD50 (G-2) sc-74460 Santa Cruz Supplier validation: Reactivity - H M R Application - WB, IP, IF and ELISA ;
RAD50 NB100-154 Novus Biologicals Supplier validation: Reactivity - Hu, Mu, Ha Application - WB, Simple Western, IP, ICC/IF ;
RBP1 (RNAPII) (D8L4Y) 14958 Cell Signaling Supplier validation: Reactivity - H M R Mk Application - WB, ChIP ;
RNAPII (4H8) 101307 Active Motif Supplier validation: Reactivity - Budding Yeast, C. elegans, Human, Mouse, Rat Application - WB, IF, ChIP, ICC ;
RPA2 sc-56770 Santa Cruz Supplier validation: Reactivity - H M R Application - WB, IP, IF, IHC(P) and ELISA ;
Vinculin (hVIN-1) V9131 Sigma Supplier validation: Reactivity - frog, chicken, mouse, canine, human, bovine, rat, turkey Application - WB, IF, IHC ;
WRNIP1 (G-2) 377402 Santa Cruz Supplier validation: Reactivity - H M R Application - WB, IP, IF, IHC(P) and ELISA ;
WRNIP1 A301-389A-T Bethyl Supplier validation: Reactivity - H Application - WB, IP, IHC ;

Eukaryotic cell lines

Policy information about cell lines and Sex and Gender in Research

Cell line source(s)	HCT116 - human colorectal carcinoma cell line was a gift from the laboratory of Martin Eilers (Würzburg, Germany) and was originally purchased from the German Collection of Microorganisms and Cell Cultures (Wiegering et al. 2015; https://doi.org/10.1158/2159-8290.CD-14-1040)<br>MEF - mouse embryonic fibroblasts were a gift from the laboratory of Martin Eilers (Würzburg, Germany). This cell line was originally isolated from E13.5 embryos (Schülein-Völk et al. 2014; https://doi.org/10.1016/j.celrep.2014.09.057).
Authentication	Human cell lines were authenticated by STR typing.
Mycoplasma contamination	Cells were tested negative for mycoplasma contamination by PCR.
Commonly misidentified lines<br>(See ICLAC register)	No commonly misidentified cell lines were used throughout this study.

ChIP-seq

Data deposition

☒ Confirm that both raw and final processed data have been deposited in a public database such as GEO.

☒ Confirm that you have deposited or provided access to graph files (e.g. BED files) for the called peaks.

Data access links<br>*May remain private before publication.*	GSE218240 (https://www.ncbi.nlm.nih.gov/geo/query/acc.cgi?acc=GSE218240)
Files in database submission	The Fastq and bedGraph files for all experiments are deposited at the GEO portal
Genome browser session<br>(e.g. UCSC)	The bedGraph files are provided via the GEO accession for vizualization using a genome browser

Methodology

Replicates	Each experiment consists of two independent replicates
Sequencing depth	At least 5000000 reads per sample
Antibodies	WRNIP1 (G-2) Santa Cruz Cat# 377402 Lot# l1516; pS2-RNAPII Active Motif Cat# 61083 Lot# 32820004
Peak calling parameters	Reads were mapped using STAR; begraph files were generated using Homer; peaks were called using Homer's FindPeaks command with default parameters
Data quality	FastQC was used to assess quality of ChIP-seq reads. Number of pS2-RNAPII peaks (regions) identified by Homer - HUWE1-WT-shCtrl: 11948, HUWE1-WT-shWRNIP1 -18936; HUWE1-CS-8377, HUWE1-CS-shWRNIP1 - 17887.
Software	Illumina bcl2fastq (version 2.20), Skewer (version 0.2.2), STAR (version 2.5.4), EdgeR (version 3.26.8), Homer (version 4.10.3)
